# DMM Outstanding Paper Prize 2025 winners: Joshua D. Ginzel and Victoria Ektnitphong

**DOI:** 10.1242/dmm.053023

**Published:** 2026-06-01

**Authors:** Rachel Hackett

**Affiliations:** The Company of Biologists, Bidder Building, Station Road, Histon, Cambridge CB24 9LF, UK

## Abstract

Disease Models & Mechanisms (DMM) is delighted to announce that the winners of the DMM Outstanding Paper Prize 2025 are Joshua D. Ginzel for their Research Article (titled ‘Nonlinear progression during the occult transition establishes cancer lethality’) and Victoria Ektnitphong for their Resources & Methods article (titled ‘An alveolus lung-on-a-chip model of *Mycobacterium fortuitum* lung infection’). The two prizes of £1000 are awarded to the first author(s) of the papers that are judged by the journal's Editors to be the most outstanding contribution to the journal that year.

## Teaser

**Summary:** DMM announces the winners of the DMM Outstanding Paper Prize for Research Articles and Resources & Methods articles published in 2025.

## Outstanding Paper Prize winner for Research Articles: Joshua D. Ginzel

**Figure:**
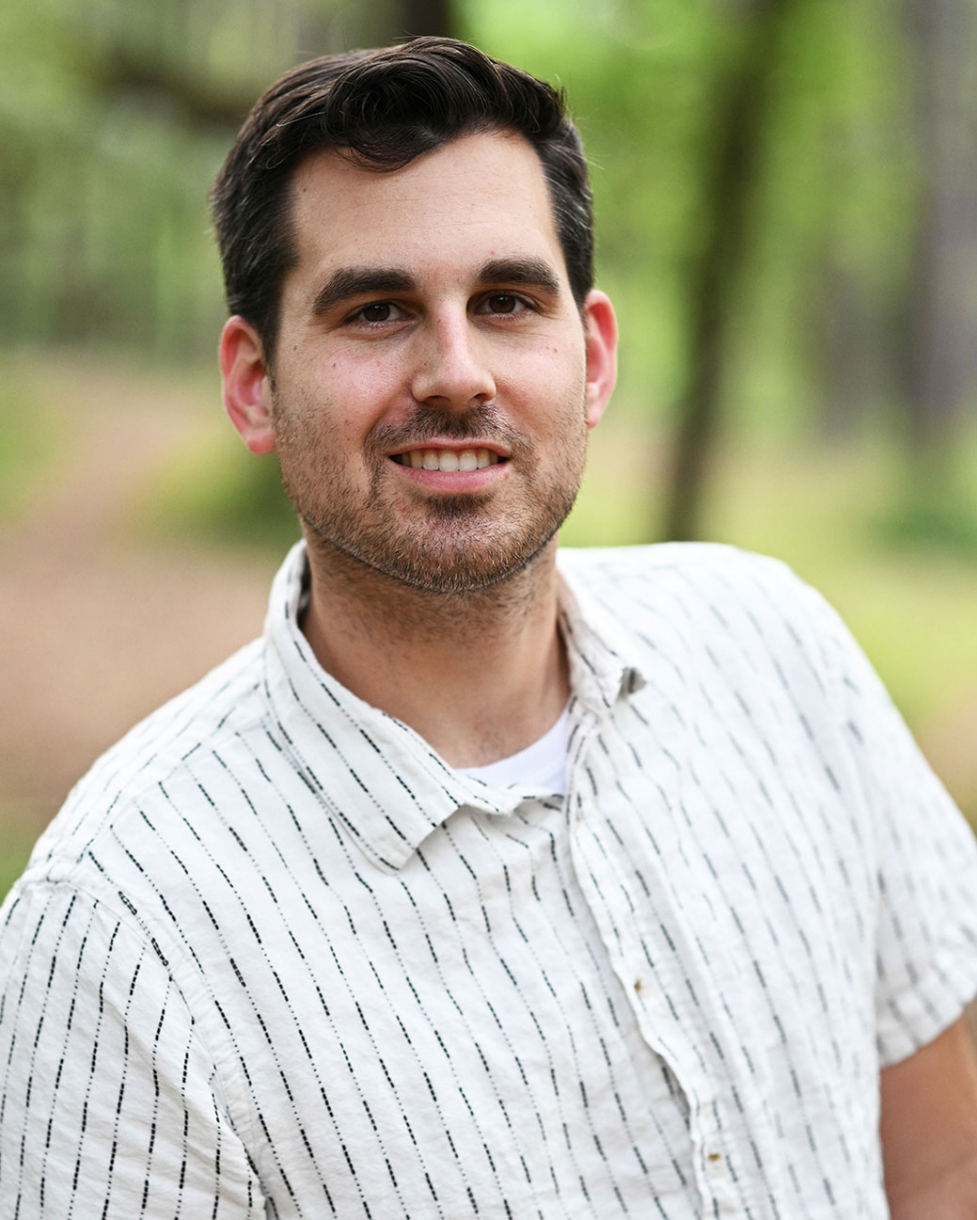
Joshua D. Ginzel

Joshua's scientific journey began at Bradley University, where he studied for a BSc in Cell and Molecular Biology. During his freshman year, a programme that rotated students through research laboratories on campus gave him his first exposure to academic science and led him to begin his undergraduate research, studying factors coordinating the development of parathyroid hormone-producing cells. During this time, Josh also worked in the laboratory of José Ramírez at the USDA Agricultural Research Service, where he studied how host–pathogen immunology in mosquitoes impacts population control efforts using entomopathogenic fungi. These experiences taught him the importance of coordinated biological systems and sparked an interest in understanding how these systems go awry in human disease.

Following graduation in 2018, Josh moved to Durham, NC, USA to pursue a PhD at Duke University in the Developmental and Stem Cell Biology programme. He joined the laboratory of Joshua Snyder in the Department of Surgery, where he became interested in the earliest, invisible stages of cancer development – the period before tumours can be detected by current clinical screening. His thesis work centred on a fundamental paradox in breast cancer: despite dramatic increases in early-stage detection through mammography, the incidence of lethal metastatic disease has not declined proportionally. Using fluorescently labelled ‘Cancer rainbow’ (Crainbow) mice developed by the Snyder laboratory, Josh combined high-throughput imaging with mathematical modelling to trace the progression of HER2-driven breast cancers from single initiating cells through to metastasis. This work demonstrated that malignant progression can occur abruptly and independently of tumour growth, identifying a class of tumours that acquire metastatic capacity before reaching a clinically detectable size. This work gave rise to a first-author Research Article published in DMM (Ginzel et al., 2025). Joshua received his PhD from the Duke University Department of Cell Biology in 2025.

In 2025, Joshua began a postdoctoral fellowship in the laboratory of Aleksandra Tata in the Division of Surgical Sciences at Duke University. His postdoctoral work investigates how pericytes in the pulmonary vasculature respond to injury and contribute to the lung repair process.

## Outstanding Paper Prize winner for Resources & Methods articles: Victoria Ektnitphong

**Figure:**
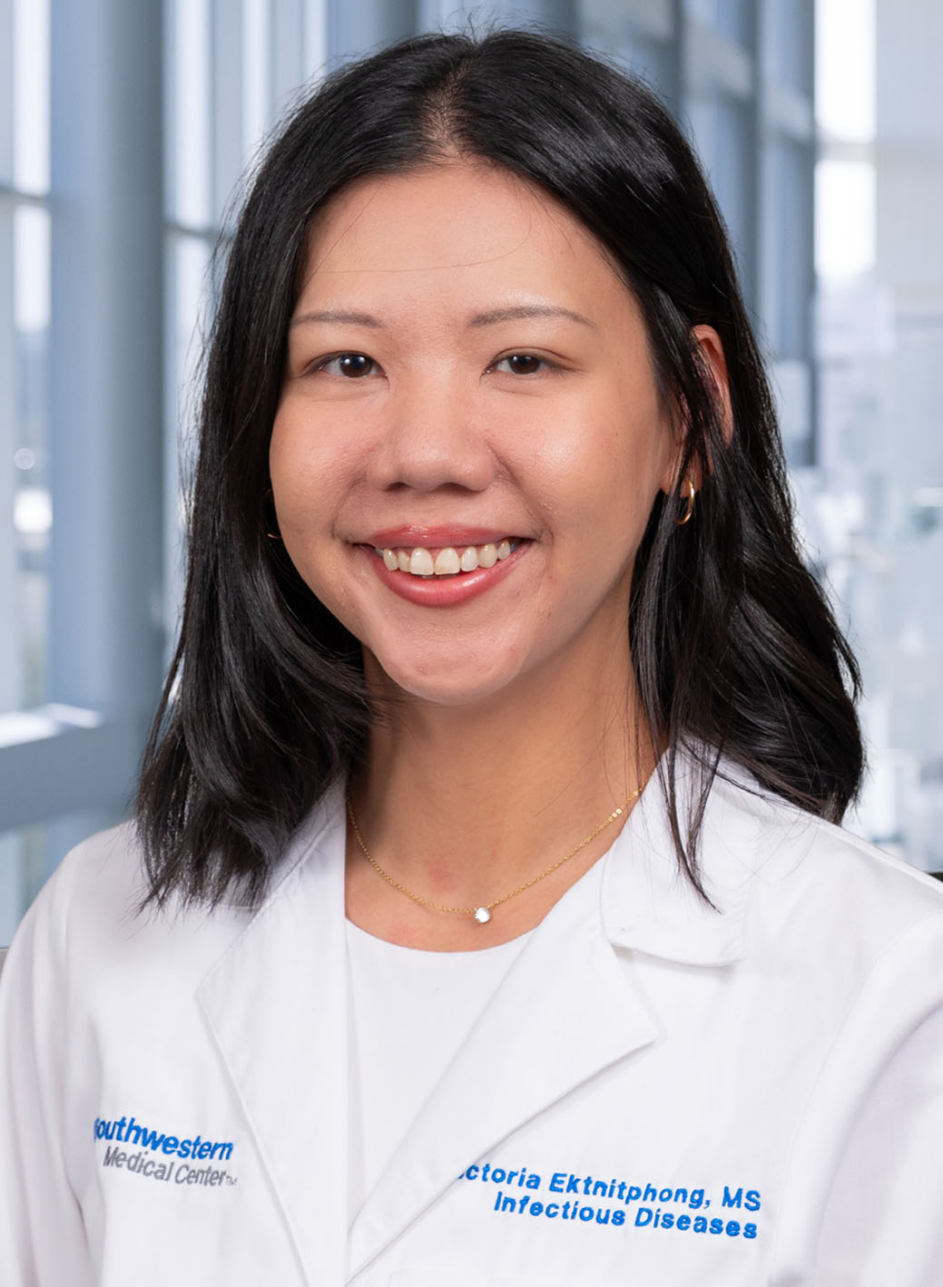
Victoria Ektnitphong

Victoria Ektnitphong is a research scientist specialising in the application of large- and small-animal models to advance antimicrobial drug development, with a particular focus on *Mycobacterium tuberculosis*. Her work integrates translational model systems to interrogate host–pathogen interactions and to support the preclinical evaluation of novel therapeutic strategies.

Victoria's research career began in 2006 at Newport Laboratories in Worthington, MN, USA, where she later continued as a research and development intern during her undergraduate studies in Biochemistry and Molecular Biology at Gustavus Adolphus College. Over 5 years, she developed a strong foundation in vaccine development through extensive work with multiple animal models, including calves, pigs, goats, rabbits and mice. Her contributions included veterinary vaccine formulation and the development of immunoassays. This early immersion in translational veterinary research shaped her focus on infectious disease and motivated her pursuit of advanced training centred on human pathogens.

In 2014, she earned her MS in Microbiology and Immunology from Colorado State University and joined the laboratory of Dr Ramesh Akkina. There, she investigated HIV pathogenesis and latency using advanced humanised mouse models. These systems enabled the development of functional human immune compartments *in vivo*, allowing her to study complex host–pathogen interactions and further strengthening her focus on physiologically relevant disease models to study infectious diseases.

Later in 2014, Victoria joined the Mycobacteria Research Laboratories at Colorado State University under Dr Anne Lenaerts and Dr Greg Robertson. Supported by major global health initiatives, including the Bill and Melinda Gates Foundation and the TB Drug Accelerator, the laboratory was focused on advancing anti-tuberculosis (TB) compounds from preclinical development toward clinical evaluation. In this role, she developed extensive expertise in murine models that recapitulate distinct stages and pathologies of TB infection, including acute (rapid, actively replicating bacteria), chronic (slowed bacterial replication) and C3HeB/FeJ (formation of caseous necrotic lesions) models. She characterised these mouse models through low- and high-dose aerosol infections, *in vivo* drug efficacy testing and comprehensive post-infection analyses. She also contributed to mechanistic studies through the isolation and characterisation of drug-resistant *M. tuberculosis* strains to elucidate drug mode-of-action and resistance pathways.

In 2019, Victoria joined the laboratory of Dr Michael Shiloh at UT Southwestern Medical Center in Dallas, TX, USA. Since joining the laboratory, she has utilised her expertise in TB models to contribute to ongoing studies of TB host–pathogen interactions while pioneering the development of a novel *in vitro* platform, the alveolus lung-on-a-chip (ALoC). Using this system, which was designed to recapitulate key features of the human alveolar microenvironment by incorporating air–liquid interface, mechanical stretch and vascular flow, she demonstrated that *Mycobacterium fortuitum* preferentially infects macrophages with limited epithelial cell involvement. She also identified early immune activation signatures through bulk RNA sequencing, including upregulation of cytokines, chemokines and SERPIN family transcripts. These findings highlight the utility of the ALoC platform in resolving early host responses to mycobacterial (Ektnitphong et al., 2025).

Her current work focuses on extending the ALoC system to additional *Mycobacterium* species, including other non-tuberculous mycobacteria and *M. tuberculosis*, with the goal of establishing physiologically relevant *in vitro* models for studying early immune responses and accelerating therapeutic evaluation.

DMM first launched its Outstanding Paper Prize in 2018, which is now one of the highlights of our editorial year. In 2025, DMM published over 105 Research Articles and Resources & Methods articles, all eligible for the Outstanding Paper Prize. Our Editors each nominated their favourite paper, resulting in a shortlist that reflects the breadth and diversity of research published in DMM. They showcase the impactful research being conducted across our community using a variety of model systems (*in vitro*, *in vivo*, *ex vivo*), techniques and approaches. You can find the full shortlist of nominated papers in Box 1.

We would like to thank everyone who published with us in 2025 for choosing DMM over the growing number of alternative publication venues in the world today. By publishing in DMM, you are demonstrating your support for a not-for-profit scientist-led journal.

Box 1. DMM Outstanding Paper Prize 2025 shortlist **Research Articles**

**GABAergic neuronal dysfunction underlies tremor in spinocerebellar ataxia 3.**

Animesh Banerjee, Moumita Chatterjee, Kah Junn Tan, Shermaine Tay, Kaibo Duan, Anand Kumar Andiappan, Shanshan Wu Howland, Yoshinori Aso and Sherry Shiying Aw.

**ETV1 is a key regulator of enteroendocrine PYY production.**

Astrid M. Baattrup, Marianne Terndrup Pedersen, Stine L. Hansen, Martti Maimets, Fiona Gribble, Frank Reimann and Kim B. Jensen.

**KNTC1 introduces segmental heterogeneity to mitochondria.**

Atsushi Tsukamura, Hirotaka Ariyama, Natsuki Hayashi, Satoko Miyatake, Satoko Okado, Sara Sultana, Ichiro Terakado, Takefumi Yamamoto, Shoji Yamanaka, Satoshi Fujii, Haruka Hamanoue, Ryoko Asano, Taichi Mizushima, Naomichi Matsumoto, Yoshihiro Maruo and Masaki Mori.

**Abca4, mutated in Stargardt disease, is required for structural integrity of cone outer segments.**

John J. Willoughby and Abbie M. Jensen.

**An *in vitro* model of the epithelial airway reveals a key function for EHF in lung homeostasis and disease.**

Laetitia Pinte, Marta Vila-Gonzalez, Eleanor C. Williams, Erika Causa, Ricardo Fradique, Tekle Pauzaite, Charlotte Passemar, Silvia Becca, Christopher Gribben, Shiqi Ye, Maha Al-Thani, Fabian Bachinger, Floris J. M. Roos, James A. Nathan, Irina Mohorianu, Andres Floto, Pietro Cicuta and Ludovic Vallier.

**Development of a genetically tailored implantation hepatocellular carcinoma model in Oncopigs by somatic cell CRISPR editing.**

Lobna Elkhadragy, Maximillian J. Carlino, Luke R. Jordan, Thomas Pennix, Nahed Ismail, Grace Guzman, Jonathan P. Samuelson, Lawrence B. Schook, Kyle M. Schachtschneider and Ron C. Gaba.

**Generation and characterization of a mouse model of Becker muscular dystrophy with a deletion of *Dmd* exons 52 to 55.**

Lucie O. M. Perillat, Tatianna W. Y. Wong, Eleonora Maino, Abdalla Ahmed, Ori Scott, Elzbieta Hyatt, Paul Delgado-Olguin, Shagana Visuvanathan, Evgueni A. Ivakine and Ronald D. Cohn.

**Glucose uptake in pigment glia suppresses Tau-induced inflammation and photoreceptor degeneration.**

Mikiko Oka, Sho Nakajima, Emiko Suzuki, Shinya Yamamoto and Kanae Ando.

**Physical and functional interaction of the ciliopathy proteins Lrrc56 and Odad3 control deployment of axonemal dyneins in vertebrate multiciliated cells.**

Nayeli G. Reyes-Nava, Chanjae Lee, Ophelia Papoulas, Juyeon Hong, Edward M. Marcotte and John B. Wallingford.

**Resources & Methods articles**

**Allograft transplantation for *Drosophila* tumor metastasis studies.**

Chaitali Khan and Nasser M. Rusan.

**Use of metabolic imaging to monitor heterogeneity of tumour response following therapeutic mTORC1/2 pathway inhibition.**

Stephanie Ling, Alex Dexter, Alan M. Race, Shreya Sharma, Gregory Hamm, Urszula M. Polanska, Rosetta Consortium Cancer Research UK, John F. Marshall, Zoltan Takats, Kevin Brindle, Mariia O. Yuneva, George Poulogiannis, Andrew D. Campbell, Owen J. Sansom, Richard J. A. Goodwin, Josephine Bunch and Simon T. Barry.

**Expanding and refining the Mammalian Phenotype Ontology to enhance disease model discovery.**

Susan M. Bello, Anna V. Anagnostopoulos, Leigh C. Carmody, Nicolas Matentzoglu and Cynthia L. Smith.

**Winner: Research Article**

**Nonlinear progression during the occult transition establishes cancer lethality.**

Joshua D. Ginzel, Henry Chapman, Joelle E. Sills, Edwin J. Allen, Lawrence S. Barak, Robert D. Cardiff, Alexander D. Borowsky, Herbert Kim Lyerly, Bruce W. Rogers and Joshua C. Snyder.

**Winner: Resources & Methods article**

**An alveolus lung-on-a-chip model of *Mycobacterium fortuitum* lung infection.**

Victoria Ektnitphong, Beatriz R. S. Dias, Priscila C. Campos and Michael U. Shiloh.
